# The Nucleotide Excision Repair Pathway Protects *Borrelia burgdorferi* from Nitrosative Stress in *Ixodes scapularis* Ticks

**DOI:** 10.3389/fmicb.2016.01397

**Published:** 2016-09-07

**Authors:** Travis J. Bourret, Kevin A. Lawrence, Jeff A. Shaw, Tao Lin, Steven J. Norris, Frank C. Gherardini

**Affiliations:** ^1^Department of Medical Microbiology and Immunology, Creighton UniversityOmaha, NE, USA; ^2^Gene Regulation Section, Laboratory of Zoonotic Pathogens, Rocky Mountain Laboratories, National Institute of Allergy and Infectious Diseases, National Institutes of HealthHamilton, MT, USA; ^3^Department of Pathology and Laboratory Medicine, McGovern Medical School, University of Texas Health Science Center at HoustonHouston, TX, USA

**Keywords:** *Borrelia*, Lyme disease, nitric oxide, oxidative stress, DNA repair

## Abstract

The Lyme disease spirochete *Borrelia burgdorferi* encounters a wide range of environmental conditions as it cycles between ticks of the genus *Ixodes* and its various mammalian hosts. Reactive oxygen species (ROS) and reactive nitrogen species (RNS) are potent antimicrobial molecules generated during the innate immune response to infection, however, it is unclear whether ROS and RNS pose a significant challenge to *B. burgdorferi in vivo*. In this study, we screened a library of *B. burgdorferi* strains with mutations in DNA repair genes for increased susceptibility to ROS or RNS *in vitro*. Strains with mutations in the methyl-directed mismatch repair gene *mutS1* are hypersensitive to killing by ROS, while strains lacking the nucleotide excision repair (NER) gene *uvrB* show increased susceptibility to both ROS and RNS. Therefore, *mutS1*-deficient and *uvrB*-deficient strains were compared for their ability to complete their infectious cycle in Swiss Webster mice and *I. scapularis* ticks to help identify sites of oxidative and nitrosative stresses encountered by *B. burgdorferi in vivo*. Both *mutS1* and *uvrB* were dispensable for infection of mice, while *uvrB* promoted the survival of spirochetes in *I. scapularis* ticks. The decreased survival of *uvrB*-deficient *B. burgdorferi* was associated with the generation of RNS in *I. scapularis* midguts and salivary glands during feeding. Collectively, these data suggest that *B. burgdorferi* must withstand cytotoxic levels of RNS produced during infection of *I. scapularis* ticks.

## Introduction

Vector-borne infectious diseases caused by viruses, bacteria, and protozoa are a major source of morbidity and mortality throughout the world and are typically transmitted by hematophagous arthropods (e.g., mosquitoes, biting flies, hemipteran bugs, and ticks). *Borrelia burgdorferi*, the etiological agent of Lyme disease, is transmitted by ticks of the genus *Ixodes* to various mammalian hosts, and is the most common vector-borne illness in North America and Europe (Lindgren and Jaenson, 2006). As of 2011, Lyme disease was listed as the sixth most common Nationally Notifiable disease in the United States with 33,097 cases, which was a 9.7% increase from 2010 (Adams et al., 2013). Recently, the Centers for Disease Control and Prevention estimated that the annual incidence of Lyme disease to be up to 329,000 in the United States (Nelson et al., 2015).

Throughout its infectious cycle *B. burgdorferi* encounters a wide range of environmental stresses including shifts in temperature, pH, osmolarity, nutrient availability, as well as ROS ($O_{2}^{•-}$, H_2_O_2_, OH^•^) and RNS (NO, ${\mathrm{NO}}_{2}^{•}$, N_2_O_3_, and ONOO^-^), and other innate and acquired immune defenses. The antimicrobial capacity of ROS and RNS is dictated by their ability to damage a variety of cellular targets including DNA, polyunsaturated fatty acids of lipid membranes, iron-containing proteins (i.e., cytochromes, Fe–S clusters), zinc metalloproteins, and sulfhydryl groups of cysteines (Henard and Vazquez-Torres, 2011). *B. burgdorferi* is highly resistant to killing by ROS produced *in vitro*, which has been attributed to (1) the limited capacity for the production of highly reactive OH^•^ by the Fenton reaction due to the lack of intracellular iron in *B. burgdorferi* cells, (2) detoxification of ROS by a small repertoire of antioxidant defenses that include a manganese-dependent superoxide dismutase (*sodA*), a coenzyme A disulfide reductase (*cdr)*, thioredoxin/thioredoxin reductase (*trxA*/*trxB*), and the neutrophil activating protein A (*napA*/*bicA*), and (3) direct scavenging of ROS by exogenous pyruvate present in BSK II culture media (Posey and Gherardini, 2000; Boylan et al., 2008; Esteve-Gassent et al., 2009; Eggers et al., 2011; Troxell et al., 2012, 2014). Additionally, clear roles for the MMR pathway and the NER pathway in the defense of *B. burgdorferi* against ROS have been demonstrated by several groups (Bourret et al., 2011; Sambir et al., 2011; Hardy and Chaconas, 2013; Troxell et al., 2014). Collectively, these factors appear to limit the toxicity of ROS in wild-type *B. burgdorferi* to the peroxidation of its cell membranes, resulting in formation of pronounced membrane blebs (Boylan et al., 2008).

In contrast to its innate resistance to ROS, *B. burgdorferi* is sensitive to killing by RNS generated *in vitro* (Bourret et al., 2011). The bactericidal activity of RNS against *B. burgdorferi* is distinct from ROS, as cell membranes are unaffected by lethal concentrations of the NO-generating compound diethylamine NONOate (DEA/NO). Rather, RNS-dependent killing of *B. burgdorferi* is the result of the *S*-nitrosylation of a large number of both free and zinc-bound cysteine thiols. The ability of antioxidant defenses that combat ROS to confer cross-protection to *B. burgdorferi* against RNS has yet to be studied. However, the NER pathway has been shown to promote resistance to RNS in *B. burgdorferi*, presumably through the repair of oxidative and/or nitrosative damage to DNA (Bourret et al., 2011; Sambir et al., 2011; Hardy and Chaconas, 2013; Troxell et al., 2014).

To date, the contribution by either ROS or RNS to the innate host defenses of *Ixodes* ticks, or mammalian hosts against *B. burgdorferi* is unclear. *SodA*-deficient strains that display an increased sensitivity to killing by ROS generated *in vitro* and *in situ*, are avirulent in a murine model of infection (Esteve-Gassent et al., 2009), while ROS-sensitive NER and MMR pathway mutants display varying degrees of infectivity that may depend on the *B. burgdorferi* strain background (Dresser et al., 2009; Lin et al., 2009; Hardy and Chaconas, 2013). Inducible nitric oxide synthase (iNOS) is the primary source of RNS in mammalian hosts, and plays a key role in innate immune defenses against a wide variety of microbial pathogens. RNS appear to be dispensable for the innate immune defenses of mice against *B. burgdorferi* as mice treated with NG- L-monomethyl arginine to suppress RNS production or iNOS-deficient C3H/HeJ mice showed similar degrees of arthritis and spirochetemia as their untreated or wild-type counterparts following infection with *B. burgdorferi* (Seiler et al., 1995; Brown and Reiner, 1999). RNS production by Ixodid ticks has been demonstrated in the dog tick *Dermacentor variabilis*, and is required for dilation of salivary ducts facilitating the release of saliva containing a cocktail of anti-inflammatory proteins and small molecules (Bhattacharya et al., 2000; Lamoreaux et al., 2000). *In silico* analysis of the *I. scapularis* genome has revealed a putative NOS homolog (*ISCW018074, nos*), suggesting that RNS may represent a significant environmental stress for *B. burgdorferi* during infection of its arthropod hosts. Supporting this hypothesis, gene silencing of dual oxidase (*duox*) or a peroxidase (*ISCW017368*) in *I. scapularis* ticks results in the disruption of the dityrosine network that crosslinks the extracellular matrix in tick midguts, and led to the concomitant increase in *nos* transcripts and NOS enzymatic activity (Yang et al., 2014). The increased expression of *nos* coincided with a reduction in the number of wild-type *B. burgdorferi* cells within the tick midguts suggesting an antibacterial role for RNS within *I. scapularis* tick midgut lumen.

To investigate the role of ROS and RNS in the host defense against *B. burgdorferi*, strains harboring mutations in DNA repair genes of the NER, MMR, and BER pathways were screened for their sensitivity to ROS and RNS produced *in vitro* by H_2_O_2_ or DEA/NO, and compared for their ability to complete their infectious cycle in Swiss–Webster mice and *I. scapularis* ticks. Additionally, we examined the salivary glands and midguts of *I. scapularis* ticks for ROS and RNS production to identify the locales of oxidative and nitrosative stresses encountered by *B. burgdorferi* during life in its arthropod host.

## Materials and Methods

### Bacterial Strains

An infectious, low-passage (<5 passages) *B. burgdorferi* strain B31 A3, as well as isogenic *uvrB* and *mutS1*-deficient strains were maintained at 34°C in BSK II medium, pH 7.6 under microaerobic conditions (3% O_2_, 5% CO_2_) in a Galaxy 170R incubator (Eppendorf, Hauppauge, NY, USA) as previously described (Bourret et al., 2011; Troxell et al., 2014). Cell density was determined by dark field microscopy. The plasmid profiles of each strain were confirmed by PCR (Bunikis et al., 2011). Construction of additional BER, MMR, and NER mutant strains is described in the Supplementary Material using primers listed in Supplementary Table S2 and strains are listed in Supplementary Table S1.

### ROS and RNS Susceptibility Assays

Late log-phase *B. burgdorferi* cultures were diluted in fresh BSK II medium, and grown at 34°C under microaerobic conditions to a cell density of ~5 × 10^7^ cells ml^-1^. Cells were pelleted by centrifugation at 3,000 rpm for 10 min, subjected to a 2 × 20 ml wash in HN buffer (20 mM NaCl, 50 mM Hepes, pH 7.6), and resuspended in HN buffer to a density of ~5 × 10^7^ cells ml^-1^. One milliliters of aliquots from the cells grown in BSK II media, or resuspended in HN buffer were transferred to 5 ml polypropylene culture tubes (Evergreen Scientific, Los Angeles, CA, USA), and incubated at 34°C under microaerobic conditions for 2 h in the presence or absence of either 2.5 mM H_2_O_2_ (Sigma, St. Louis, MO, USA), or 1.0 mM diethylamine NONOate (Cayman Chemical, Ann Arbor, MI, USA). Following treatment, serial dilutions of cells were prepared in HN buffer, and were plated on semi-solid BSK II media. Plates were incubated at 34°C under microaerobic conditions for 7–14 days, and colony forming units (CFUs) were enumerated. Percent survival was determined by dividing CFUs from the 2 h timepoint samples by the CFUs from the 0 h timepoint.

### Mouse-Tick Cycle of Infection

Mouse studies were conducted at the University of Nebraska at Kearney and the Rocky Mountain Laboratories, and all protocols approved by each institution’s Animal Care and Use Committee. The Rocky Mountain Laboratories are accredited by the International Association for Assessment and Accreditation of Laboratory Animal Care (AAALAC). Six- to eight-week-old Swiss Webster mice (Charles River Laboratories, Wilmington, MA, USA) or RML mice, an outbred strain of Swiss Webster mice maintained at RML since 1937, were used for infection studies. Individual mice were inoculated via subcutaneous injection with target doses of 1 × 10^3^ or 1 × 10^5^ spirochetes/mouse. Three weeks following inoculation, ear punch biopsy samples were taken from each mouse and cultured in 10 ml BSK II medium at 34°C under microaerobic conditions for 7–21 days to assess infection with *B. burgdorferi*.

The ability of *B. burgdorferi* strains to be acquired by *I. scapularis* ticks from infected mice was determined by feeding approximately 100 larval ticks to repletion on individual mice. Next, individual ticks were washed in 70% EtOH, and then homogenized in 0.2 ml BSK II medium with a motorized pestle (Kimble Chase, Vineland, NJ, USA). Serial dilutions of tick homogenates were prepared in HN buffer, and samples were plated on semi-solid BSK II plates supplemented with amphotericin B (2.5 μg ml^-1^) and rifampicin (10 μg ml^-1^). Homogenization and plating of ticks was conducted after ticks had fed to repletion, or after molting to nymphs (62–81 days post-feeding). Plates were incubated at 34°C under microaerobic conditions for 7–21 days, and CFUs were enumerated.

Transmission of *B. burgdorferi* strains from infected ticks to naïve mice was determined by feeding cohorts of 10–20 *B. burgdorferi*-infected nymphs on RML mice. Recovered ticks were homogenized, and samples were cultured in 10 ml of BSK II to determine infectivity. Mice were euthanized 3 weeks after being fed upon by infected nymphs. Ears, bladders, and ankle joints were collected from each mouse, and inoculated in 10 ml BSK II medium. Tissue samples were incubated at 34°C under microaerobic conditions for 7–21 days, and monitored for the presence of spirochetes by dark field microscopy.

### Detection of ROS and RNS in Tick Salivary Glands and Midguts

Reactive nitrogen species produced in tick tissues were detected with the fluorescent probe 4,5-diaminofluorescein diacetate (DAF-2) purchased from Cayman Chemical (Ann Arbor, MI, USA). DAF-2 reacts with NO in the presence of oxygen to produce triazolofluorescein (DAF-2T). Briefly, salivary glands and midguts were dissected from unfed adult *I. scapularis* female ticks, or from adult female ticks fed to repletion on New Zealand White rabbits. Salivary glands and midguts were rinsed extensively in PBS, and transferred to individual 1.5 ml microcentrifuge tubes with 25 μM DAF-2 for 10 min at RT. Following incubation, tick tissues were placed in individual pools of ~10 μL PBS on glass microscope slides, and imaged using a Nikon Eclipse E800 epifluorescence microscope (Nikon USA, Melville, NY, USA) by digital interference contrast (DIC) microscopy or fluorescence microscopy using a FITC filter (Ex_λ_ = 495 nm, Em_λ_ = 519 nm).

### RNA Isolation and RT-qPCR Analysis of *I. scapularis* Genes

Total RNA was isolated from *I. scapularis* larvae and nymphs following feeding on *B. burgdorferi*-infected Swiss Webster mice. Briefly, salivary glands and midguts were dissected from fed nymphs, and transferred to individual 1.5 ml microcentrifuge tubes with 1 ml RNAzol (Sigma-Aldrich, St. Louis, MO, USA), while pools of five blood-fed larvae were homogenized in 1 ml RNAzol using a motorized pestle. RNA was isolated per the manufacturer’s instructions, and RNA concentrations were measured using a TAKE3 plate and a Cytation 5 multi-mode plate reader (Biotek, Winooski, VT, USA). DNA contamination was eliminated from RNA samples using Turbo-free DNase (Life Technologies, Carlsbad, CA, USA) per the manufacturer’s instructions. Following DNase treatment, cDNA was generated from 250 ng of total RNA using the iScript cDNA synthesis kit (Bio-Rad Laboratories, Hercules, CA, USA). Quantitative reverse transcription PCR (RT-qPCR) reactions were prepared for tick cDNA samples using Bullseye EvaGreen qPCR master mix (MIDSCI, St. Louis, MO, USA) and oligonucleotide primers for *act*, *nos*, *duox*, and *salp25D* (Narasimhan et al., 2007; Yang et al., 2014). The RT-qPCR reactions were performed using a CFX Connect Real-time PCR Detection System (Bio-Rad) with cycling conditions of 95°C for 10 s, 59°C for 20 s, and 72°C for 30 s followed by melt-curve analysis to determine the efficiency and specificity of the qPCR reactions. The relative expression of selected genes normalized to *act* was determined using the 2^-ΔΔ^*^C^*^T^ method (Livak and Schmittgen, 2001).

### NOS Expression and Nitrotyrosine Formation in Tick Salivary Glands and Midguts

Salivary glands and midguts harvested from *I. scapularis* nymphs fed on mice, were fixed and permeabilized using Fix and Perm Kit (Thermo Fisher Scientific, Waltham, MA, USA) per the manufacturer’s instructions. Tick tissues were incubated in PBS in the presence of BSA (10 mg ml^-1^), and rabbit serum as a negative control or with a 1:100 dilution of a universal NOS antibody (Thermo Fisher) for 3 h. Tissues were then subjected to 3 × 0.5 ml washes in PBS, and then incubated with a 1:200 dilution of the Goat anti-Rabbit IgG secondary antibody Alexa Fluor 488 conjugate (Life Technologies) and a 1:400 dilution of DAPI (Thermo Fisher) for an additional 3 h at RT. Tissues were washed as described above, and then visualized by fluorescent microscopy using the Blue channel (Ex_λ_ = 355 nm, Em_λ_ = 433 nm) and the Green channel (Ex_λ_ = 480 nm, Em_λ_ = 517 nm) of the ZOE Flourescent Cell Imager (Bio-Rad). Similarly, salivary glands and midguts were incubated in PBS in the presence of rabbit serum or an anti-nitrotyrosine polyclonal antibody (Thermo Fisher) diluted 1:100 for 3 h at RT. Salivary glands were washed in PBS as described above, followed by incubation with a 1:200 dilution of a Goat anti-Rabbit IgG secondary antibody Alexa Fluor 647 conjugate (Life Technologies), and a 1:400 dilution of DAPI for an additional 3 h. Tissues were washed with PBS and visualized using the Blue channel (Ex_λ_ = 355 nm, Em_λ_ = 433 nm) and the Red channel (Ex_λ_ = 556 nm, Em_λ_ = 615 nm) of the ZOE Flourescent Cell Imager (Bio-Rad).

## Results

### The NER and MMR Systems Protect *B. burgdorferi* from Oxidative and Nitrosative Stresses

**FIGURE 1:**
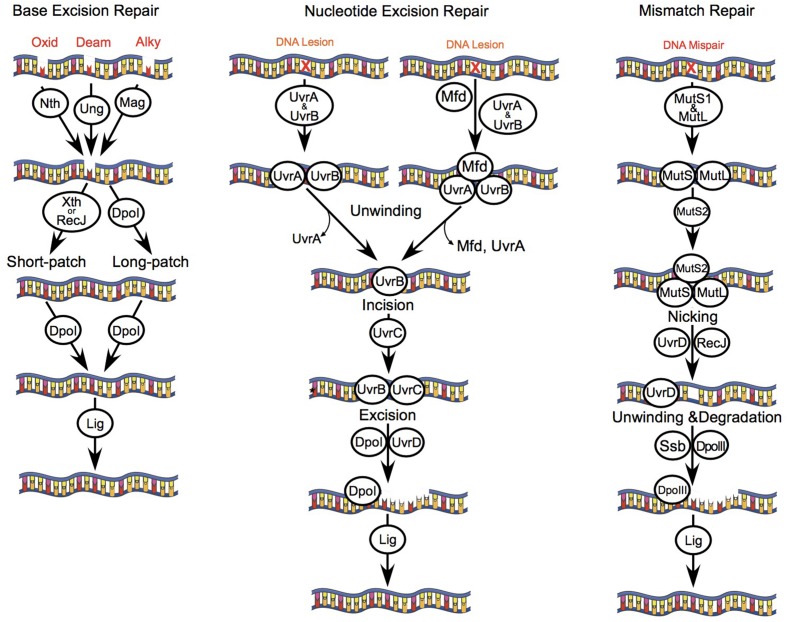
**DNA repair pathways of *B. burgdorferi*.** *B. burgdorferi* encodes genes of several DNA repair pathways including: BER, NER, and MMR. The BER pathway consists of monofunctional glycosylases including uracil DNA glycosylase (Ung, BB0053) and 3-methyladenine glycosylase (Mag, BB0422); a bifunctional glycosylase/endonuclease III (Nth, BB0745); and an exonuclease (XthA, BB0534). Glycosylases recognize and remove DNA bases that are oxidized (8-oxoguanine), alkylated, or deaminated leaving an apurinic or apyrimidinic site (AP site). AP endonucleases (Nth and/or XthA) cleave one nucleotide for short-patch BER, or 2–13 nucleotides for long-patch BER. The combined action of ssDNA exonuclease (RecJ, BB0254), DNA polymerase I (Dpol, BB0548), and DNA ligase (Lig, BB0552) repair the DNA lesion. The NER pathway removes bulky chemically induced lesions, lesions induced by UV light, or otherwise unrecognized DNA lesions that distort the DNA helix using the UvrABC endonuclease complex (UvrBA, BB0836-837; UvrC, BB0457). UvrA and UvrB form a dimer that scans the genome for DNA lesions, or is actively recruited to DNA lesions by the transcription coupling factor Mfd (BB0627). Following recognition of a lesion, UvrA dissociates from UvrB. Next, UvrB forms a dimer with UvrC that cuts a 12 base-pair segment of DNA, which is excised by DNA helicase II (UvrD, BB0344). The resulting gap in host DNA is then filled by Dpol and Lig. The MMR pathway in *B. burgdorferi* consists of the mismatch repair proteins MutS1 (BB0797), MutS2 (BB0098), and MutL (BB0211), but lacks a homolog for the weak endonuclease MutH. MutS1 binds to mismatched or damaged bases in the DNA strand, and then forms a complex with MutL. Typically, MutH binds to hemi-methylated regions of DNA, and is activated following contact with MutL. However, the *B. burgdorferi* MutL homolog encodes a putative endonuclease domain that may have activity that compensates for the lack of MutH, as has been described for *Thermus thermophilus* (Shimada et al., 2013). MutL is therefore activated upon binding to MutS1, resulting in a cut and displacement of the damaged DNA strand. Next, RecJ and UvrD excise the damaged strand of DNA. The single stranded binding protein (SSB, BB0114) protects the exposed segment of ssDNA, which is then repaired by DNA polymerase III (DpoIII, BB0438) and Lig. The cellular role of MutS2 in *B. burgdorferi* is unknown, although it may function as an inhibitor of homologous recombination, or the repair of oxidized DNA bases as described for *Helicobacter pylori* (Wang et al., 2005)

**FIGURE 2:**
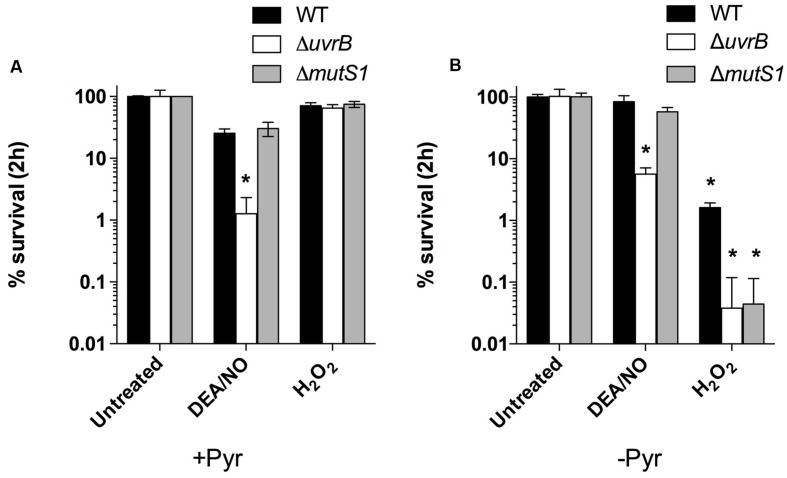
**The NER and MMR pathways contribute to *B. burgdorferi* resistance to ROS and RNS produced chemically *in vitro*.** Wild-type *B. burgdorferi* strain B31 A3 and the isogenic DNA repair mutants *ΔuvrB::aadA* and *ΔmutS1::aadA* were grown to a cell density of 5 × 10^7^ cells ml^-1^ in BSK II medium under microaerobic (3% O_2_, 5% CO_2_) conditions. *B. burgdorferi* strains were treated with 1.0 mM diethylamine NONOate (DEA/NO) or 2.5 mM H_2_O_2_ for 2 h at 34°C in either BSK-II medium **(A)** or in HN (25 mM Hepes, 100 mM NaCl) buffer **(B)**. Following incubation, cells were diluted in fresh BSK II medium and plated on solid BSK II media. Data represent the mean CFUs ± SD of six replicates collected from three separate experiments. ^∗^*P* < 0.05 compared to wild-type controls using a two-way ANOVA.

Previous studies exploring the effects of ROS and RNS on *B. burgdorferi* suggest that their antimicrobial activity relies on lipid peroxidation (ROS), or damage to free and zinc-bound cysteine thiols (RNS) rather than from genotoxicity arising from oxidative DNA damage (Boylan et al., 2008; Bourret et al., 2011). The lack of DNA damage in *B. burgdorferi* cells exposed to lethal concentrations of ROS- and RNS-generating compounds has been attributed primarily to genes of the MMR and NER pathways, although *B. burgdorferi* also encodes genes for the BER pathway (**Figure 1**). The NER pathway has been the most extensively studied in *B. burgdorferi*, and strains harboring mutations in *uvrA*, *uvrB*, *uvrC*, or *uvrD* display increased sensitivity to killing by multiple DNA damaging agents, including various ROS and RNS, mitomycin C, and UV light (Bourret et al., 2011; Sambir et al., 2011; Hardy and Chaconas, 2013; Troxell et al., 2014). Additionally, the MMR pathway gene *mutS1* has an essential role in defense against oxidative DNA damage following exposure of *B. burgdorferi* to H_2_O_2_ (Troxell et al., 2014). However, the roles of the other MMR genes *mutL* and *mutS2*, as well as the BER genes *nth* and *xthA*, in defense against ROS/RNS have yet to be explored. Therefore, we tested the susceptibility of a library of BER, MMR, and NER pathway mutant strains of *B. burgdorferi* to the NO donor DEA/NO, and H_2_O_2_. Consistent with previous studies (Hardy and Chaconas, 2013; Troxell et al., 2014), strains with mutations in the NER genes *uvrB* or *uvrC* were 25-fold and 15-fold more sensitive to killing by 1 mM DEA/NO in BSK II medium compared to a wild-type *B. burgdorferi* B31 A3 strain, while MMR-deficient strains (B31 A3: Δ*mutL*, Δ*mutS*, and Δ*mutS2*; 5A18N: *mutL*^-^, *mutS*^-^, and *mutS2*^-^), and BER-deficient strains (B31 A3: Δ*xthA*, 5A4: Δ*nth*) survived at levels indistinguishable from their wild-type counterparts (**Figure 2A**; Supplementary Figure S1). All of the strains tested were resistant to killing by 2.5 mM H_2_O_2_ when challenged in BSK II media, with the exception of the *B. burgdorferi* B31 A3 *ΔmutS2* strain (**Figure 2A**; Supplementary Figure S1). The resistance of *B. burgdorferi* to H_2_O_2_ in BSK II media is due in part to the presence of 7 mM sodium pyruvate, which is a potent scavenger of H_2_O_2_, and other ROS and RNS. When the wild-type, Δ*uvrB*, and Δ*mutS1 B. burgdorferi* strains were exposed to H_2_O_2_ or DEA/NO in pyruvate-free HN buffer, the *uvrB*- and *mutS1*-deficient *B. burgdorferi* strains were ~40-fold more sensitive to killing by H_2_O_2_ than their wild-type counterpart (**Figure 2B**). In contrast, all strains showed similar levels of susceptibility to DEA/NO in both HN buffer and BSK II media, suggesting that increased susceptibility of the *uvrB*-deficient mutant to DEA/NO may be due to nitrosative stress or oxidative stress from higher oxides of NO including ${\mathrm{NO}}_{2}^{•}$. Collectively, these data indicate that the NER pathway plays a preeminent role in combatting DNA damage induced by both ROS and RNS, while MMR pathway primarily contributes to resistance to oxidative DNA damage.

### DNA Repair Genes *uvrB* and *mutS1* Are Dispensable for Infection of Mice

*Borrelia burgdorferi* may encounter potentially damaging ROS and RNS during infection of mammalian hosts. This is supported by the fact that strains lacking the *Borrelia* oxidative stress regulator (BosR), which coordinates the expression of antioxidant defenses, are hypersensitive to oxidative stress, and are attenuated in murine model of infection (Hyde et al., 2009; Ouyang et al., 2011). Additionally, *sodA*-deficient *B. burgdorferi*, which are more readily killed by ROS than wild-type strains when challenged *in vitro*, are also unable to infect mice through intradermal inoculation (Esteve-Gassent et al., 2009). Wild-type *B. burgdorferi* cells are sensitive to killing by RNS produced *in vitro*, however, a contribution by RNS to host defenses against *B. burgdorferi* has yet to be demonstrated. Previous studies revealed a slight decrease in infectivity for NER-deficient *B. burgdorferi* strains, and MMR-deficient strains (*mutL*^-^, *mutS1*^-^, and *mutS2*^-^) following inoculation of C3H/HeN mice (Dresser et al., 2009; Lin et al., 2009; Hardy and Chaconas, 2013). Because of their differential susceptibility to ROS and RNS, *uvrB*-deficient and *mutS1*-deficient *B. burgdorferi* B31 A3 strains were assessed for their ability to complete their infectious cycle in Swiss Webster or RML mice, and *I. scapularis* ticks. Both the *uvrB*-deficient and *mutS1*-deficient *B. burgdorferi* strains successfully infected mice when inoculated at doses of either 10^3^ or 10^5^ spirochetes/mouse, respectively (**Table 1**). Spirochetes were isolated from ears, knee joints, and bladders of infected mice, suggesting that *B. burgdorferi* may not encounter significant amounts of ROS or RNS following artificial infection of mice and subsequent dissemination, or that alternative defenses (e.g., SodA) may sufficiently protect *B. burgdorferi* against oxidative and nitrosative stresses despite mutations in the NER or MMR pathways.

**Table 1:** Infection of mice with wild-type and DNA-repair deficient *B. burgdorferi.*

Strain	Number of infected mice/number of mice analyzed	Ears	Joints	Bladder	Total	% Infected Tissues
**Dose: 1 × 10^3^ spirochetes/mouse**						
B31 A3 wild-type	6/6	6/6	6/6	6/6	18/18	100%
B31 A3 *ΔuvrB*	5/6	5/6	5/6	5/6	15/18	83.30%
B31 A3 *ΔmutS1*	5/6	5/6	5/6	5/6	15/18	83.30%
**Dose: 1 × 10^5^ spirochetes/mouse**						
B31 A3 wild-type	12/12	12/12	12/12	12/12	36/36	100%
B31 A3 *ΔuvrB*	12/12	11/12	12/12	12/12	35/36	95.80%
B31 A3 *ΔmutS1*	12/12	12/12	12/12	11/12	35/36	95.80%

### The NER Pathway Promotes the Colonization of *I. scapularis* Ticks by *B. burgdorferi*

**FIGURE 3:**
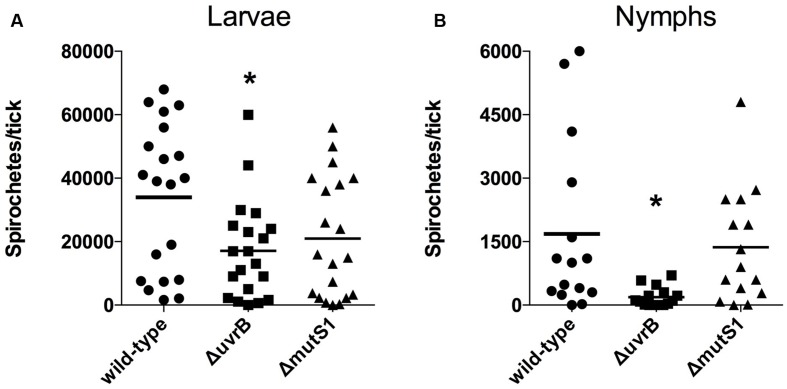
**The NER pathway promotes survival of *B. burgdorferi* in *I. scapularis* ticks.** The number of viable *B. burgdorferi* spirochetes in ticks after feeding on Swiss-Webster mice infected with wild-type, *ΔuvrB::aadA*, or *ΔmutS1::aadA B. burgdorferi* strains was determined prior to the molt at 12–18 days post-feeding **(A),** and after molting to nymphs at 62–81 days **(B)**. Individual ticks were homogenized, and serial dilutions were plated on solid BSK II medium. The number of *B. burgdorferi* CFUs from individual ticks are shown, along with the mean number of spirochetes per tick. ^∗^*P* < 0.05 compared to wild-type controls using a one-way ANOVA.

It is unclear whether *B. burgdorferi* encounters potentially lethal concentrations of ROS or RNS during infection of *I. scapularis* ticks. A recent study has highlighted a crucial role for a peroxidase (ISCW017368) and dual oxidase (Duox) in the formation of a network of dityrosine complexes in the extracellular matrix of the tick midgut during feeding, which is hypothesized to restrict pathogenic microbes to the midgut (Yang et al., 2014). However, it was unclear from this study whether *B. burgdorferi* encounters significant amounts of oxidative or nitrosative stresses during infection of *I. scapularis* ticks. Inhibition of *duox* expression by RNAi led to increased expression of *nos* and NOS enzymatic activity, along with a lower number of *B. burgdorferi* cells in the tick midgut, which was attributed to RNS toxicity. As demonstrated in **Figure 2**, the *mutS1*-deficient *B. burgdorferi* strain is hypersensitive to ROS, and the *uvrB*-deficient strain is hypersensitive to both ROS and RNS produced *in vitro*. Therefore, we compared the ability of *I. scapularis* larvae to acquire wild-type, *ΔuvrB* and *ΔmutS1 B. burgdorferi* strains following feeding on infected Swiss Webster mice. The acquisition of *B. burgdorferi* strains by *I. scapularis* ticks was assessed by homogenizing individual larval ticks after feeding to repletion (3–6 days), and plating serial dilutions on solid BSK media. Larvae fed on mice infected with wild-type *B. burgdorferi* harbored an average of 33,900 spirochetes/tick compared to 17,128 and 20,953 spirochetes/tick for mice infected with the Δ*uvrB* and Δ*mutS1 B. burgdorferi* strains, respectively (**Figure 3A**). Following the molt of larvae to nymphs, individual fed ticks were assessed for colonization by the wild-type, Δ*uvrB*, and Δ*mutS1 B. burgdorferi* strains. Ticks fed on mice infected with the wild-type *B. burgdorferi* averaged 1,685 spirochetes/tick, while those fed on mice infected with the Δ*mutS1* strain had 1,367 spirochetes/tick (**Figure 3B**). In contrast, samples taken from ticks that fed on mice infected with the *uvrB*-deficient strain harbored significantly lower numbers of spirochetes (189 spirochetes/tick) compared to wild-type controls. Collectively, these data indicate that the NER pathway protects *B. burgdorferi* against DNA damage in the tick as spirochetes are acquired in the blood meal and through the molt of larvae to nymphs.

Next, the ability of infected ticks to transmit wild-type, *ΔuvrB*, or Δ*mutS1 B. burgdorferi* spirochetes to naïve mice was tested. Groups of mice were infested with 10 or 20 *B. burgdorferi*-infected nymphs, and assessed for infection 3 weeks following the completion of tick feeding. The wild-type *B. burgdorferi* B31 A3 strain was successfully transmitted to 6/6 mice following infestation with 10 or 20 infected *I. scapularis* nymphs, with positive cultures for spirochetes in isolated ears, joints, and bladders (**Table 2**). Similarly, all of the mice infested with nymphs carrying the Δ*mutS1 B. burgdorferi* strain were positive for infection, although the bladder isolated from one mouse was free of spirochetes. Despite a nearly 10-fold reduction in numbers of *uvrB*-deficient *B. burgdorferi* carried by *I. scapularis* nymphs (**Figure 3**), the transmission of the *ΔuvrB* strain was observed in 7/8 mice. This is consistent with the reportedly small inoculum of tick-derived *B. burgdorferi* spirochetes required for the establishment of infection in naïve mice (Kasumba et al., 2016). Collectively, these data indicate that the MMR and NER pathways are not required for the successful transmission of *B. burgdorferi* from *I. scapularis* nymphs to murine hosts.

**Table 2:** Transmission of *B. burgdorferi* strains from *I. scapularis* nymphs to naïve mice.

Strain	Number of ticks/mouse	Number of infected mice/number of mice analyzed	Culture-positive tissues				
			Ears	Joints	Bladder	Total infected sites	% Infected tissues
B31 A3 wild-type	10	3/3	3/3	3/3	3/3	9/9	100%^∗^
	20	3/3	3/3	3/3	3/3	9/9	100%
B31 A3 *ΔuvrB*	10	5/5	5/5	5/5	5/5	15/15	100%
	20	2/3	2/3	2/3	2/3	6/9	66.60%
B31 A3 *ΔmutS1*	20	3/3	3/3	3/3	2/3	8/9	100%

*^∗^Two of five mice infested with I. scapularis nymphs carrying the B. burgdorferi B31 A3 wild-type strain died prior to testing for transmission.*

### ROS and RNS Are Produced in *I. scapularis* Salivary Glands and Midguts

**FIGURE 4:**
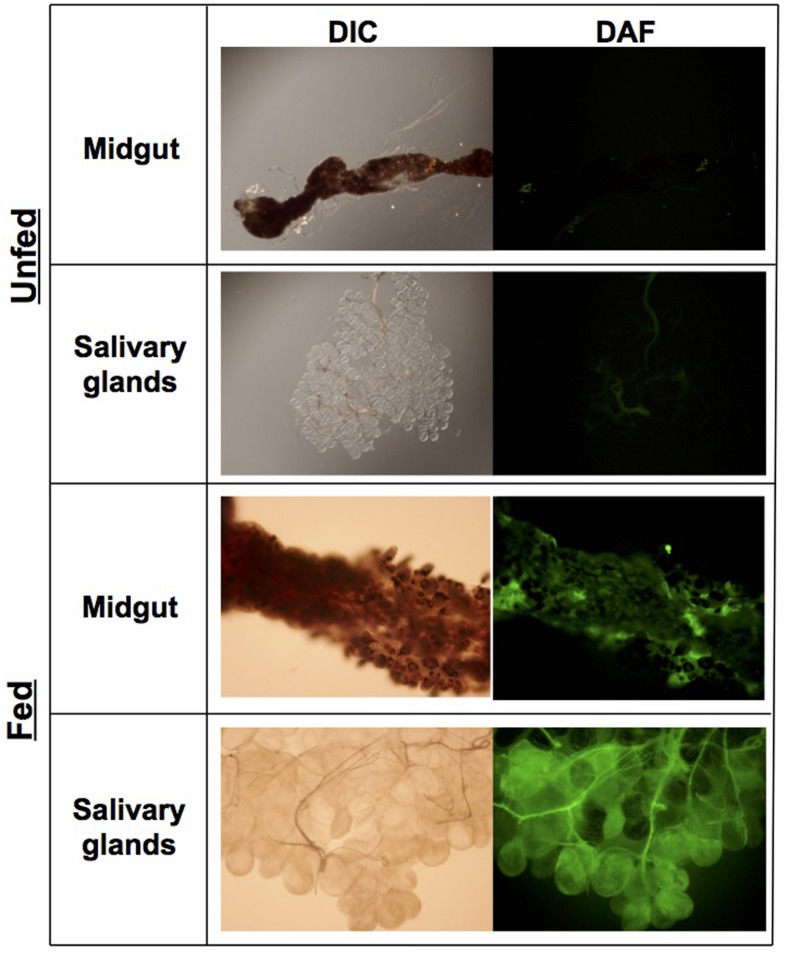
**RNS production is induced during feeding of *I. scapularis* adults.** Midguts and salivary glands were harvested from unfed ticks and ticks fed on rabbits. Organs were rinsed in PBS and subsequently incubated in PBS + 25 μM DAF-2 (5,6-diaminoflorescein diacetate) for 10 min. Images were collected using digital interference contrast (DIC) microscopy, or immunofluorescent microscopy using a FITC filter (Ex_λ_ = 495 nm, Em_λ_ = 519 nm).

The locales of oxidative and nitrosative stresses encountered by *B. burgdorferi* during its infectious cycle have remained ambiguous. Within its mammalian hosts, *B. burgdorferi* may encounter potentially harmful ROS and RNS at the tick bite site produced by professional phagocytes including neutrophils and macrophages. However, components of *I. scapularis* saliva including the peroxiredoxin Salp25D may protect *B. burgdorferi* by directly detoxifying ROS and RNS, while other components may antagonize the production of ROS and RNS at the tick bite site (Narasimhan et al., 2007). The fact that the ROS- and RNS-sensitive Δ*uvrB B. burgdorferi* strain showed a survival defect in *I. scapularis* ticks, while the ROS-sensitive Δ*mutS1* strain was found in similar numbers to wild-type controls (**Figure 3**), suggests that *B. burgdorferi* encounters potentially harmful concentrations of RNS during infection of its arthropod host. The fluorescent indicator dye 4,5-diaminofluorescein diacetate (DAF-2) reacts with RNS generated by the reaction of NO with $O_{2}^{•-}$ (e.g., N_2_O_3_, ONOO^-^) to yield the highly fluorescent derivative triazolofluorescein (DAF-2T). Midguts and salivary glands harvested from unfed, adult *I. scapularis* females incubated with DAF-2 failed to produce a DAF-2T fluorescent signal (**Figure 4**). In contrast, midguts and salivary glands harvested from engorged *I. scapularis* females yielded strong fluorescent signals, which were indicative of RNS production (**Figure 4**). Additionally, saliva samples were collected from adult *I. scapularis* females following feeding to repletion on New Zealand white rabbits and tested for nitrite (NO_2_^-^) levels using the fluorogenic probe 2,3-diaminonapthalene (DAN), which reacts with N_2_O_3_ to form 1(H)-naphthotriazole. Tick saliva samples contained high levels of NO_2_^-^ ranging from 0.19 to 1.34 mM (Supplementary Figure S2), which was consistent with the apparent robust production of RNS observed in **Figure 4**. Together, these data indicate that RNS production is induced in *I. scapularis* ticks in response to feeding, and that *B. burgdorferi* must withstand a highly nitrosative environment in both the midguts and salivary glands of fed ticks.

### Expression of Genes Involved in ROS and RNS Production in *I. scapularis* Larvae and Nymphs

**FIGURE 5:**
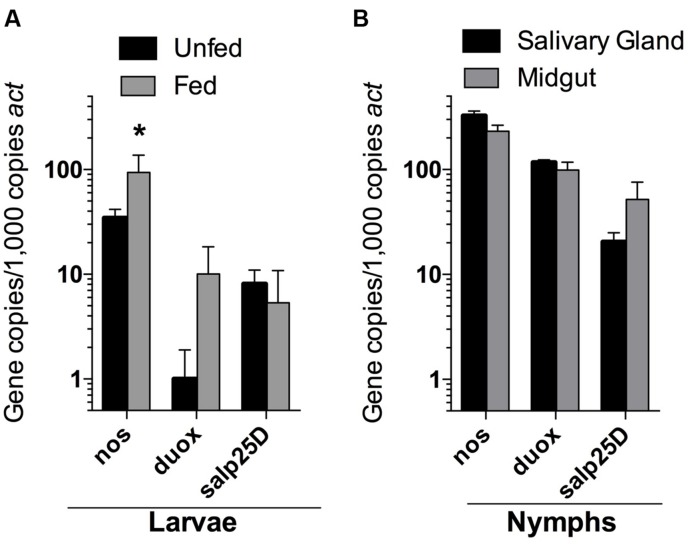
**Expression of *nos, duox*, and *salp25D* in *I. scapularis* larvae and nymphs.** RNA isolated from whole *I. scapularis* larvae **(A)**, and salivary glands and midguts of nymphs **(B)** were analyzed by RT-qPCR for the transcription of *nos, duox, and salp25D.* The expression levels of each gene were normalized using actin (*act*) as a reference gene. Data represent the mean ± SD of 4–8 biological replicates. ^∗^*P* < 0.05 compared to unfed controls using a two-way ANOVA.

**FIGURE 6:**
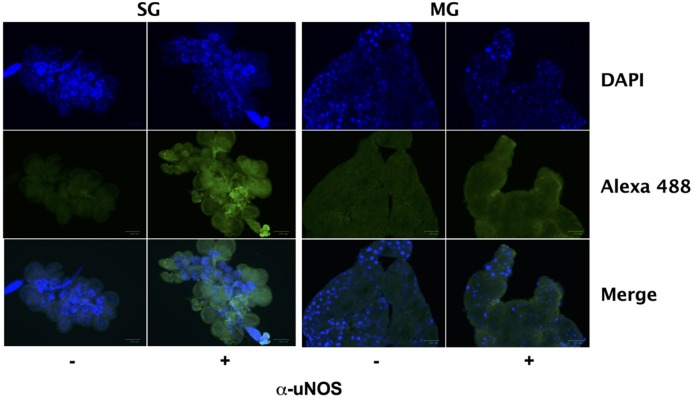
**Nitric oxide synthase is expressed during feeding of *I. scapularis* ticks.** Salivary glands (SG) and midguts (MG) harvested from *I. scapularis* nymphs fed on Swiss Webster mice were probed with rabbit serum (-) or with a universal NOS (uNOS) antibody (+) followed by incubation with an Alexa Fluor 488 secondary antibody and DAPI.

Despite the seemingly robust production of RNS in *I. scapularis* salivary glands and midguts during feeding (**Figure 4**), *B. burgdorferi* may also encounter substantial amounts of ROS and RNS in *I. scapularis* ticks following the molt of larvae to nymphs as evidenced by reduced numbers of *uvrB*-deficient *B. burgdorferi* (**Figure 3**). The sources of ROS needed to react with NO to produce the nitrosative environment detected in *I. scapularis* ticks likely include (1) the breakdown of the oxygen-rich blood meal in the midgut, and (2) the enzymatic activity of ROS-generating enzymes such as Duox and NADPH oxidase. In addition to production of NO by NOS, RNS may also be produced through the acidification of NO_2_^-^ in the tick midgut (pH 6.8) during feeding (Bhattacharya et al., 2000; Lamoreaux et al., 2000; Yang et al., 2000; Yang et al., 2014). To better understand the impact of feeding on RNS production, the transcript levels of *nos* and *duox* was measured in RNA samples harvested from *I. scapularis* larvae prior to feeding and following feeding to repletion on Swiss Webster mice. The expression of *nos* increased significantly (~3-fold) in response to feeding, while *duox* expression was elevated 10-fold (**Figure 5A**). Similar transcript levels of the peroxiredoxin *salp25D* were observed in both unfed and fed *I. scapularis* larvae. The increased expression of both *nos* and *duox* was consistent with the increased production of RNS observed in *I. scapularis* ticks fed on rabbits (**Figure 4**). Next, RNA was isolated from salivary glands and midguts of *I. scapularis* nymphs following feeding on Swiss Webster mice, and the expression of *nos*, *duox*, and *salp25D* were compared by RT-qPCR. The expression of both *nos* and *duox* were similar in both the salivary glands and midguts of *I. scapularis* nymphs, while the expression of *salp25D* was ~2.5-fold higher in tick midguts compared to salivary glands (**Figure 5B**), which was consistent with the previously reported pattern of *salp25D* expression (Narasimhan et al., 2007). Additionally, immunofluorescence using a universal NOS antibody demonstrated that NOS was expressed in both salivary glands and midguts of fed *I. scapularis* nymphs (**Figure 6**). Collectively, these data are consistent with roles for Duox and NOS in the robust production of RNS detected in *I. scapularis* midguts and salivary glands (**Figure 4**).

### Assessment of the Nitrooxidative Environment in Tick Midguts and Salivary Glands

**FIGURE 7:**
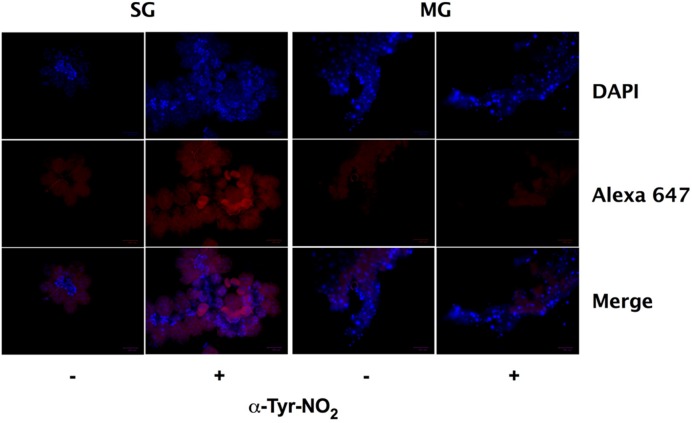
**Nitrotyrosine formation in salivary glands during feeding of *I. scapularis* nymphs.** Salivary glands (SG) and midguts (MG) harvested from *I. scapularis* nymphs fed on Swiss Webster mice were probed with rabbit serum (-) or with an anti-nitrotyrosine antibody (+) followed by incubation with an Alexa Fluor 647 secondary antibody and DAPI.

Previously, the enzymatic activity of ROS-generating enzymes Duox, NADPH oxidase, and peroxidase (ISCW017368) were linked to the formation of dityrosine complexes in the *I. scapularis* midgut during feeding (Yang et al., 2014). In addition to ROS, highly oxidative RNS (e.g., ONOO^-^ and ${\mathrm{NO}}_{2}^{•}$) can also catalyze dityrosine formation, as well as the formation of nitrotyrosine. Nitrotyrosine is a covalent post-translational modification of proteins that is commonly used as a marker for RNS arising from the autoxidation of NO produced by iNOS or from the reaction of peroxidases with NO_2_^-^, and has been linked to the antiplasmodial immunity of *Anopheles gambiae* (Ischiropoulos, 1998; Oliveira et al., 2012). Within the tick salivary glands and midguts, $O_{2}^{•-}$ produced by Duox and NADPH oxidase may react with NO generated by NOS to form substantial amounts of ONOO^-^. To further explore this possibility, nitrotyrosine formation was assessed by immunofluorescent microscopy of salivary glands and midguts harvested from engorged *I. scapularis* nymphs after feeding on Swiss Webster mice. Extensive nitrotyrosine formation was observed in *I. scapularis* salivary glands, while midguts failed to show any signs of nitrotyrosine formation (**Figure 7**). These data suggest that highly oxidative RNS are present in the salivary glands of feeding ticks, while the RNS produced in the midgut are not capable of inducing the formation of nitrotyrosine.

## Discussion

Hematophagous arthropods, including Ixodid ticks, have complex innate immune defenses within the midgut to combat microbial pathogens. For example, both the mosquito *A. gambiae* and *I. scapularis* ticks form a peritrophic membrane in the midgut at the onset of feeding along with a ROS-induced dityrosine network that limits the access of pathogens to the midgut epithelium (Shen and Jacobs-Lorena, 1998; Devenport et al., 2004; Kumar et al., 2010; Yang et al., 2014). The dityrosine complexes appear to limit the expression of NOS by walling off microbial invaders from the midgut epithelium, thereby preventing the induction of a proinflammatory response. Gene silencing of *duox* in *I. scapularis* ticks leads to the upregulation of *nos* and increased NOS activity, which corresponds to lower numbers of *B. burgdorferi*, indicating a bactericidal effect of tick-borne RNS (Yang et al., 2014). The data presented herein indicate that *B. burgdorferi* encounters substantial concentrations of RNS while residing in the *I. scapularis* midgut. This is supported by the significant reduction in *uvrB*-deficient *B. burgdorferi* numbers in *I. scapularis* ticks as blood-fed larvae progress through the molt to nymphs. We observed that the production of RNS is induced in both salivary glands and midguts of *I. scapularis* ticks at the onset of feeding. Previously, it has been noted that NO generated in *D. variabilis* stimulated dilation of the salivary duct promoting the efficient secretion of the sialome (Bhattacharya et al., 2000; Lamoreaux et al., 2000). The role of RNS generated in the salivary gland may extend beyond the dilation of salivary ducts of ticks. For example, RNS are potent vasodilators that may increase blood flow at the tick bite site. The presence of millimolar concentrations of NO_2_^-^ measured in the saliva harvested from *I. scapularis* adults suggests that high concentrations of NO are delivered to the bite site (Supplementary Figure S2).

The sites of oxidative and nitrosative stresses encountered by *B. burgdorferi* during its infectious cycle have remained ambiguous. For example, *B. burgdorferi* strains harboring mutations in *sodA* are attenuated in a murine model of infection, and show enhanced susceptibility to killing by ROS generated chemically *in vitro*, or *in situ* by activated macrophages and neutrophils (Esteve-Gassent et al., 2009; Troxell et al., 2012). Furthermore, mutations of *bosR* increase the susceptibility of *B. burgdorferi* to oxidative stress (Hyde et al., 2009, 2010). In contrast*, uvrB*- and *mutS*-deficient strains exhibiting hypersensitivity to low concentrations (0.1–0.5 mM) of authentic H_2_O_2_ when challenged in HN buffer (**Figure 2** and (Troxell et al., 2014)) are fully capable of infecting Swiss Webster mice at doses ranging from 10^3^–10^5^ spirochetes per mouse (**Table 1**). The discrepancy between the ability of *sodA* mutants and DNA repair mutants to infect mice may be reconciled by the fact that the absence of *sodA* results in *B. burgdorferi* cells with (1) a single 20 kDa isoform of BosR that may lack its Zn^2+^ cofactor due to oxidation by $O_{2}^{•-}$ and (2) higher molecular weight isoforms of NapA that possibly result from a ROS-scavenging role for NapA (Esteve-Gassent et al., 2009). Because BosR-dependent gene expression appears to require Zn^2+^ (Boylan et al., 2003; Ouyang et al., 2011), the absence of SodA likely disrupts BosR-dependent induction of the RpoS-OspC relay, which may account for the inability of the *sodA* mutant strain to infect mice (Esteve-Gassent et al., 2009; Esteve-Gassent et al., 2015). Conversely, SodA activity is likely sustained in the *uvrB*- and *mutS*-deficient *B. burgdorferi* strains, thereby maintaining the BosR-dependent induction of genes required for mammalian infection. Further investigations will be required to determine the mechanism underlying the attenuated phenotype of *sodA*-deficient *B. burgdorferi*, and what role ROS play in mammalian host defenses against *B. burgdorferi*.

Similar to the case of ROS, there does not appear to be a significant role for RNS in mammalian host defenses against *B. burgdorferi*. This is supported by the ability of RNS-hypersensitive Δ*uvrB B. burgdorferi* strain used in our study to infect Swiss Webster mice at identical doses as wild-type controls. The fact that the Δ*uvrB B. burgdorferi* strain was able to successfully infect mice and to be acquired by *I. scapularis* larvae suggests that *B. burgdorferi* does not encounter high enough levels of ROS or RNS capable of overwhelming their antioxidant/antinitrosative defenses (e.g., SodA, NER, MMR) during infection of mice. In contrast, *uvrB*-deficient *B. burgdorferi* were found in substantially lower numbers during infection of *I. scapularis* larvae and nymphs. The decrease in *uvrB*-deficient *B. burgdorferi* numbers may be attributed to the increase in oxidative and nitrosative stresses in the tick midgut. However, the fact that the ROS-sensitive *ΔmutS1* strain survived at levels identical to wild-type *B. burgdorferi* suggests a more prominent role for RNS, rather than ROS as a challenge for *B. burgdorferi* while residing in *I. scapularis* ticks.

The production of RNS in both the salivary glands and midguts of ticks is induced by feeding. Our study does not address whether the presence of *B. burgdorferi* induces a higher level of RNS production compared to uninfected controls, as was previously reported for ROS-generating enzymes (Yang et al., 2014). While we did observe expression of NOS, the production of RNS, and the formation of nitrotyrosine in *I. scapularis* following feeding, it is not yet clear whether RNS production is sustained as larval progress through the molt to nymphs. The reduction in *uvrB*-deficient *B. burgdorferi* cells following the molt of larvae to nymphs suggests that RNS production may continue following feeding. Rather than playing a direct role in host defenses, RNS production may be required for tick development as larvae progress through the molt, as has been described in *Drosophila melanogaster* (Yu et al., 2010).

This study also revealed that *I. scapularis* salivary glands generate large amounts of RNS during feeding resulting in the extensive formation of nitrotyrosine (**Figure 7**). Previously, NOS enzymatic activity was linked to the dilation of salivary ducts in the dog tick *D. variabilis* (Bhattacharya et al., 2000; Lamoreaux et al., 2000), which likely enhances the flow of tick saliva into the bite site. Interestingly, very little is known about the amount of RNS produced within various tick tissues. We observed salivary NO_2_^-^ concentrations reaching greater than 1 mM, which is likely due to a high level of NOS activity and RNS production within the salivary glands (Supplementary Figure S2). Other hematophagous arthropods such as *Rhodnius prolixus*, utilize salivary RNS and/or NO-containing heme molecules referred to as nitrophorins to promote vasodilation and anti-hemastasis at the bite site (Ribeiro and Walker, 1994; Champagne et al., 1995; Nussenzveig et al., 1995; Andersen et al., 2005; Araujo et al., 2009). This strategy is likely conserved in *I. scapularis* ticks, and further studies will be required to characterize the effects of tick-borne salivary RNS on the innate host defenses of mammalian hosts at the bite site. Despite the high levels of RNS production in the salivary gland, *uvrB*-deficient *B. burgdorferi* were successfully transmitted to naïve mice by *I. scapularis* nymphs (**Table 2**). This suggests that despite the reduced numbers in the tick midgut, surviving Δ*uvrB B. burgdorferi* cells are able to migrate from the midgut to the salivary glands, and be deposited into the bite site. This may be possible due to the highly transient nature of *B. burgdorferi* in the *I. scapularis* salivary gland, or may be due to some protection afforded by the antioxidant defenses of the tick that are prominent components of the sialome.

With the exception of SodA, still relatively little is known about the antioxidant defenses of *B. burgdorferi*. For example, CoA is the major low molecular weight thiol in *B. burgdorferi*, and has been shown to react with H_2_O_2_ *in vitro* (Boylan et al., 2006). The *cdr*-encoded CoA reductase ulitizes NADH to maintain CoA in a reduced state. However, the contribution of the CoA/Cdr system to the antioxidant defenses of *B. burgdorferi* is unclear, as strains harboring mutations in *cdr* are as resistant to H_2_O_2_ stress as wild-type controls (Eggers et al., 2011). This may be explained by the manner in which the susceptibility assays were conducted, where pyruvate in the BSK II media may have effectively scavenged H_2_O_2_, thereby protecting what was presumably a ROS-sensitive *B. burgdorferi* strain, as was the case for the ROS-sensitive Δ*uvrB* and Δ*mutS1* strains shown in **Figure 2**. Additional putative antioxidant defenses of *B. burgdorferi* include thioredoxin/thioredoxin reductase (*trxA*/*trxB*), methionine disulfide reductase (*bb0340*), and *napA* (*bicA*/*dpsA*). The role of NapA in antioxidant defense is dubious. Though *napA* expression is induced by BosR in cells exposed to ROS, *napA*-deficient strains have been reported to be recalcitrant to ROS killing (Li et al., 2007; Wang et al., 2012). Further investigations will be needed to clarify the roles of the various antioxidant defenses in protecting *B. burgdorferi* from ROS generated *in vivo*.

Still very little is known about how *B. burgdorferi* combats RNS, or whether its described or putative antioxidant defenses afford cross protection. Previously, we showed that *B. burgdorferi* is sensitive to killing by RNS because of nitrosative damage to free and zinc-bound cysteine thiols (Bourret et al., 2011). Interestingly, NapA was one of the proteins identified as a target of *S*-nitrosylation in RNS-treated *B. burgdorferi*. Additionally, it is known that NapA is required for the persistence of *B. burgdorferi* in *I. scapularis* ticks following the acquisition of spirochetes from the blood meal (Li et al., 2007). This observation, combined with the data presented herein, raise the possibility that NapA may be a crucial component of the antinitrosative defenses of *B. burgdorferi* by scavenging potentially lethal RNS before they damage important cytosolic targets like DNA and metabolic enzymes. Additional studies will be required to decipher the relevance of *S*-nitrosylation of NapA to *B. burgdorferi* persistence in ticks. The CoA/Cdr pathway may also contribute to the antinitrosative defenses of *B. burgdorferi*. A recent study revealed that *S*-nitrosylated CoA (SNO-CoA) regulates protein *S*-nitrosylation in yeast (Anand et al., 2014). Therefore, the CoA/Cdr system may regulate *S*-nitrosylation, or directly scavenge RNS in *B. burgdorferi*. In addition to CoA/Cdr, the TrxA/TrxB system may also contribute to the defense of *B. burgdorferi* against RNS toxicity through denitrosylase activity associated with thioredoxin (Benhar et al., 2008). In this scenario, rather than detoxify RNS directly, TrxA would reduce *S*-nitrosylated Cys thiols in *B. burgdorferi* proteins, and then be recycled through the enzymatic activity of TrxB. To date, there have been no formal investigations on the cellular role of TrxA/TrxB in *B. burgdorferi*.

Our study has provided important insights into the role of DNA repair pathways in defending *B. burgdorferi* against ROS and RNS toxicity both *in vitro* and *in vivo*. The NER pathway appears to play a prominent role in repairing DNA damage in *B. burgdorferi* challenged with ROS and RNS *in vitro* (Bourret et al., 2011; Troxell et al., 2014), and in the harsh milieu of the tick midgut. Additionally, the MMR protein MutS1 plays a crucial role in protecting *B. burgdorferi* from oxidative DNA damage induced by H_2_O_2_ (**Figure 2**). The susceptibility of the Δ*mutS1* strain to H_2_O_2_ was identical to the *uvrB*-deficient *B. burgdorferi* strain. Subsequent experiments will be needed to determine whether there is an additive effect of knocking out components of both the MMR and NER pathway on ROS sensitivity in *B. burgdorferi*. Alternatively, crosstalk between the MMR and NER pathway may occur following ROS-induced DNA damage. In this case, recognition of oxidative DNA lesions by MutS1 may be required for the recruitment of NER proteins necessary to repair the DNA damage. Surprisingly, a role for the BER in defense against ROS or RNS was not apparent as Δ*nth* and Δ*xthA* strains showed similar levels of sensitivity to DEA/NO as their wild-type parental controls (Supplementary Figure S1). It is possible that the efficient repair of ROS or RNS-induced DNA lesions by the NER and MMR pathways mask the contribution of BER pathway components. Furthermore, it is unclear whether BER genes are expressed under the conditions used in our study.

In summary, our investigation has provided evidence that *B. burgdorferi* encounters substantial amounts of RNS during infection of *I. scapularis* ticks. It remains unclear whether tick-borne RNS have a significant effect on the ability of wild-type *B. burgdorferi* to complete their infectious cycle. However, the fact that *B. burgdorferi* proteins are highly susceptible to RNS-induced post-translational modifications raises the possibility that this pathogen may exploit these tick-borne antimicrobial effectors as signals for coordinating virulence gene expression. This possibility will require investigations into the effects of RNS on global gene expression in *B. burgdorferi*, along with the identification of redox-active proteins in *B. burgdorferi* that may impact metabolism, gene expression, and overall virulence of this important pathogen. Moreover, RNS may also play important roles in the antimicrobial defenses of Ixodid ticks against a variety of important zoonotic pathogens.

## Author Contributions

TB, JS, and KL designed, analyzed, executed experiments. TL and SN provided mutant strains of *B. burgdorferi* as part of an ongoing collaboration. FG senior author and laboratory where experiments were performed and wrote manuscript with TB and KL.

## Conflict of Interest Statement

The authors declare that the research was conducted in the absence of any commercial or financial relationships that could be construed as a potential conflict of interest. The reviewer AV-T declared a past supervisory role with one of the authors TB to the handling Editor, who ensured that the process met the standards of a fair and objective review.

## Abbreviations

BER: base excision repair
H_2_O_2_: hydrogen peroxide
MMR: methyl-directed mismatch repair
NER: nucleotide excision repair
NO: nitric oxide
${\mathrm{NO}}_{2}^{•}$: nitrogen dioxide
N_2_O_3_: nitrous anhydride
OH^•^: hydroxyl radical
ONOO^-^: peroxynitrite
ROS: reactive oxygen species
RNS: reactive nitrogen species
$O_{2}^{•-}$: superoxide
