# Supplementary material for: The Nucleotide Excision Repair Pathway Protects Borrelia burgdorferi from Nitrosative Stress in Ixodes scapularis Ticks
Source: Front Microbiol. 2016 Sep 7;7:1397. doi: 10.3389/fmicb.2016.01397 (PMC5013056; doi:10.3389/fmicb.2016.01397)
Supplement: Supplementary file 1 [file Data_Sheet_1.DOCX]

**Supplementary material**

**Materials and Methods**

*Generation of DNA repair-deficient B. burgdorferi strains.*

Deletions of *mutL*, *mutS2*, and *xthA* in the *B. burgdorferi* B31 A3 background were achieved using a previously described mutagenesis strategy ([Bourret *et al.*, 2011](#_ENREF_1)). Briefly, 0.5 – 1 kb regions upstream and downstream of the genes of interest were amplified by PCR from wild-type *B. burgdorferi* B31 A3 DNA, and the streptomycin resistance cassette (*aadA*) under the control of a *flgB* promoter (*flgB_P_-aadA*) was amplified from pKFSS1 using La Taq polymerase (Takara Bio Inc., Madison, WI) and primers with engineered restriction sites (Table S2). The PCR products for the upstream and downstream regions of the gene of interest were cloned along with the *flgB_P_-aadA* PCR product into pPCR-Script Cam SK(+) (Agilent Technologies, Inc., Cedar Creek, TX) to produce the pCm::*ΔmutL::aadA,* pCm::*ΔmutS2::aadA* and pCm::*ΔxthA::aadA* (Table S1). Twenty micrograms of pCm::*ΔmutL::aadA,* pCm::*ΔmutS2::aadA,* or pCm::*ΔxthA::aadA* was electroporated into *B. burgdorferi* B31 A3 and transformants were selected by plating on BSK II medium supplemented with 40 µg ml^-1^ streptomycin. PCR was used to verify *ΔmutL::aadA*, *ΔmutS2::aadA,* and *ΔxthA::aadA* clones using the primers listed in Table S2. The presence of the linear and circular plasmids of *B. burgdorferi* B31 A3 were assayed using primers and PCR conditions previously described ([Bunikis *et al.*, 2011](#_ENREF_2)).

*Measurement of I. scapularis salivary nitrite.*

Adult, female *I. scapularis* ticks were placed in a capsule attached to a New Zealand white rabbit and allowed to feed to repletion ([Burgdorfer, 1984](#_ENREF_3)). Saliva samples were collected from five adult, female *I. scapularis* using a pulled capillary tube. Nitrite levels (NO_2_^-^) in tick saliva samples were determined using the fluorogenic probe 2,3-diaminonapthalene (DAN), which reacts with dinitrogen trioxide (N_2_O_3_) to form 1(H)-naphthotriazole as previously described ([Misko *et al.*, 1993](#_ENREF_9)). Briefly, saliva samples diluted 1:50 in H_2_O were mixed with 10 μl of DAN (0.05 mg ml^-1^) in 0.62 M and incubated at 20°C for 10 min. Reactions were terminated with 5 μl of 2.8 N NaOH and fluorescence was measured using Victor3V 1420 multilabel reader (PerkinElmer, Waltham, MA). Dilutions of NaNO_2_ were used to produce a standard curve to determine salivary NO_2_^-^ concentrations.

| **Table S1. Strains and plasmids** |  |  |  |
| --- | --- | --- | --- |
| Strains |  | Reference |  |
| *Borrelia burgdorferi* B31 A3 wild-type |  | ([Elias *et al.*, 2002](#_ENREF_5)) |  |
| *Borrelia burgdorferi* B31 A3 *ΔuvrB::aadA* |  | ([Bourret *et al.*, 2011](#_ENREF_1)) |  |
| *Borrelia burgdorferi* B31 A3 *ΔuvrC::aadA* |  | This study |  |
| *Borrelia burgdorferi* B31 A3 *ΔmutL::aadA* |  | This study |  |
| *Borrelia burgdorferi* B31 A3 *ΔmutS::aadA* |  | ([Troxell *et al.*, 2014](#_ENREF_12)) |  |
| *Borrelia burgdorferi* B31 A3 *ΔmutS2::aadA* |  | This study |  |
| *Borrelia burgdorferi* B31 A3 *ΔxthA::aadA* |  | This study |  |
| *Borrelia burgdorferi* B31, 5A4 wild-type |  | ([Purser & Norris, 2000](#_ENREF_11)) |  |
| *Borrelia burgdorferi* B31, 5A4 *Δnth::aacC1* |  | ([Dresser *et al.*, 2009](#_ENREF_4)) |  |
| *Borrleia burgdorferi* B31, 5A18N, wild-type |  | ([Kawabata *et al.*, 2004](#_ENREF_7)) |  |
| *Borrleia burgdorferi* B31, 5A18N *mutL*-, T10TC315 |  | ([Lin *et al.*, 2012](#_ENREF_8)) |  |
| *Borrleia burgdorferi* B31, 5A18N *mutS*-, T10TC062 |  | ([Lin *et al.*, 2012](#_ENREF_8)) |  |
| *Borrleia burgdorferi* B31, 5A18N *mutS2*-,T10TC437 |  | ([Lin *et al.*, 2012](#_ENREF_8)) |  |
| Plasmids |  |  |  |
| pKFSS1 |  | ([Frank *et al.*, 2003](#_ENREF_6)) |  |
| pPCR-Script Cam SK(+) |  | Agilent |  |
| pCm::*ΔxthA::aadA* |  | This study |  |
| pCm::*ΔmutL::aadA* |  | This study |  |
| pCm::*ΔmutS2::aadA* |  | This study |  |

| **Table S2: Oligonucleotides** | | |  |
| --- | --- | --- | --- |
| Primer Name |  | Sequence (5'-3') | Reference |
| aadA-ClaI-F |  | ACTGTAATCGATTACCCGAGCTTCAAGGAA | This study |
| aadA-SacII-R |  | ACTGTACCGCCGTATTTGCCGACTACCTTG | This study |
| aadA-EcoRI-F |  | ACTGTAGGATCCTACCCGAGCTTCAAGGAA | This study |
| aadA-EcoRI-R |  | ACTGTAGGATCCTATTTGCCGACTACCTTG | This study |
| xthA-KpnI-F |  | ACTAGCGGTACCGTAACATATGGTTGGAACCT | This study |
| xthA-EcoRI-R |  | ACTAGCGAATTCCATAAATCCTCTCTAACTAAAG | This study |
| xthA-EcoRI-F |  | ACTAGCGAATTCAGGGAGGAGGCTAGATTGAA | This study |
| xthA-SacI-R |  | ACTAGCGAGCTCTTCTTCATTAAGTGAGCCCA | This study |
| mutL-KpnI-F |  | ACTGTAGGTACCACTCACAAATACCCAAGAAG | This study |
| mutL-ClaI-R |  | ACTGTAATCGATTTCCCTTAATATTGAACATG | This study |
| mutL-SacII-F |  | ACTGTACCGCGGAAACCAGAGTTCCCATGATG | This study |
| mutL-SacI-R |  | ACTGTAGAGCTCATGAGTCGGGTGCTCTAACC | This study |
| mutS2-KpnI-F |  | ACTGTAGGTACCATCTTCGCTTTAAGGGAATG | This study |
| mutS2-ClaI-R |  | ACTGTAATCGATATCTTGTTCATCTTGCAT | This study |
| mutS2-SacII-F |  | ACTGTACCGCGGATGGAGGGTCTGGAAAAACC | This study |
| mutS2-SacI-R |  | ACTGTAGAGCTCAGCTCCATCCCTTCCTTAAG | This study |
| act-F |  | ACATGCTATCGTGGGTGACGAAGT | ([Narasimhan *et al.*, 2007](#_ENREF_10)) |
| act-R |  | TGTGGTGCCAGATCTTCTCCATGT | ([Narasimhan *et al.*, 2007](#_ENREF_10)) |
| nos-RT-F |  | TGAAGCACATGGAGAACGAG | ([Yang *et al.*, 2014](#_ENREF_13)) |
| nos-RT-R |  | GTAGTTGGGCTTGAGGTGATAG | ([Yang *et al.*, 2014](#_ENREF_13)) |
| duox-RT-F |  | CGAGAAGCAGCGCTACGA | ([Yang *et al.*, 2014](#_ENREF_13)) |
| duox-RT-R |  | CATCAACACTTCCGAGGAGA | ([Yang *et al.*, 2014](#_ENREF_13)) |
| salp25D-RT-F |  | CCTTTCCCCAACTTCACC | ([Narasimhan *et al.*, 2007](#_ENREF_10)) |
| salp25D-RT-R |  | GTCCATGGTTGTTCGGAG | ([Narasimhan *et al.*, 2007](#_ENREF_10)) |
| *The underlined sequence denotes the restriction endonuclease site of the primer. | | |  |

**

**Figure S1. Susceptibility of DNA repair mutant strains to ROS and RNS.** The viability of DNA repair mutant strains constructed in *B. burgdorferi* B31 A3 (A), 5A4 (B) and 5A18N (C) parental strain backgrounds was tested when left untreated (UT) or treated with 1.25 mM DEA/NO (NO) or 2.5 mM H_2_O_2_ for 2 h at 34°C. Percent survival was determined by dividing CFUs from the 2 h timepoint samples by the CFUs from the 0 h timepoint.

**Figure S2. Detection of NO metabolites in *I. scapularis* saliva.** Saliva samples were collected from five adult, female *I. scapularis* ticks using a pulled capillary tube **(A)**. Nitrite levels (NO_2_^-^) in tick saliva samples were determined using the fluorogenic probe 2,3-diaminonapthalene (DAN), which reacts with dinitrogen trioxide (N_2_O_3_) to form 1(H)-naphthotriazole **(B)**. The concentration of NO_2_^-^ is displayed for each of the 5 tick saliva samples tested, along with the mean.

**References**

Bourret, T.J., J.A. Boylan, K.A. Lawrence & F.C. Gherardini, (2011) Nitrosative damage to free and zinc-bound cysteine thiols underlies nitric oxide toxicity in wild-type Borrelia burgdorferi. *Mol Microbiol* **81**: 259-273.

Bunikis, I., S. Kutschan-Bunikis, M. Bonde & S. Bergstrom, (2011) Multiplex PCR as a tool for validating plasmid content of Borrelia burgdorferi. *Journal of microbiological methods* **86**: 243-247.

Burgdorfer, W., (1984) The New Zealand white rabbit: an experimental host for infecting ticks with Lyme disease spirochetes. *The Yale journal of biology and medicine* **57**: 609-612.

Dresser, A.R., P.O. Hardy & G. Chaconas, (2009) Investigation of the genes involved in antigenic switching at the vlsE locus in Borrelia burgdorferi: an essential role for the RuvAB branch migrase. *PLoS pathogens* **5**: e1000680.

Elias, A.F., P.E. Stewart, D. Grimm, M.J. Caimano, C.H. Eggers, K. Tilly, J.L. Bono, D.R. Akins, J.D. Radolf, T.G. Schwan & P. Rosa, (2002) Clonal polymorphism of Borrelia burgdorferi strain B31 MI: implications for mutagenesis in an infectious strain background. *Infection and immunity* **70**: 2139-2150.

Frank, K.L., S.F. Bundle, M.E. Kresge, C.H. Eggers & D.S. Samuels, (2003) aadA confers streptomycin resistance in Borrelia burgdorferi. *Journal of bacteriology* **185**: 6723-6727.

Kawabata, H., S.J. Norris & H. Watanabe, (2004) BBE02 disruption mutants of Borrelia burgdorferi B31 have a highly transformable, infectious phenotype. *Infection and immunity* **72**: 7147-7154.

Lin, T., L. Gao, C. Zhang, E. Odeh, M.B. Jacobs, L. Coutte, G. Chaconas, M.T. Philipp & S.J. Norris, (2012) Analysis of an ordered, comprehensive STM mutant library in infectious Borrelia burgdorferi: insights into the genes required for mouse infectivity. *PloS one* **7**: e47532.

Misko, T.P., R.J. Schilling, D. Salvemini, W.M. Moore & M.G. Currie, (1993) A fluorometric assay for the measurement of nitrite in biological samples. *Analytical biochemistry* **214**: 11-16.

Narasimhan, S., B. Sukumaran, U. Bozdogan, V. Thomas, X. Liang, K. DePonte, N. Marcantonio, R.A. Koski, J.F. Anderson, F. Kantor & E. Fikrig, (2007) A tick antioxidant facilitates the Lyme disease agent's successful migration from the mammalian host to the arthropod vector. *Cell host & microbe* **2**: 7-18.

Purser, J.E. & S.J. Norris, (2000) Correlation between plasmid content and infectivity in Borrelia burgdorferi. *Proceedings of the National Academy of Sciences of the United States of America* **97**: 13865-13870.

Troxell, B., J.J. Zhang, T.J. Bourret, M.Y. Zeng, J. Blum, F. Gherardini, H.M. Hassan & X.F. Yang, (2014) Pyruvate protects pathogenic spirochetes from H2O2 killing. *PloS one* **9**: e84625.

Yang, X., A.A. Smith, M.S. Williams & U. Pal, (2014) A dityrosine network mediated by dual oxidase and peroxidase influences the persistence of Lyme disease pathogens within the vector. *The Journal of biological chemistry* **289**: 12813-12822.
